# The Role of Social Media Use in Peer Bullying Victimization and Onset of Anxiety Among Indonesian Elementary School Children

**DOI:** 10.3389/fpsyg.2021.635725

**Published:** 2021-04-28

**Authors:** Dian Veronika Sakti Kaloeti, Rouli Manalu, Ika Febrian Kristiana, Mariola Bidzan

**Affiliations:** ^1^Faculty of Psychology, Diponegoro University, Semarang, Indonesia; ^2^Faculty of Social and Political Sciences, Diponegoro University, Semarang, Indonesia; ^3^Institute of Psychology, University of Gdansk, Gdansk, Poland

**Keywords:** social media, bullying victimization, gender, anxiety, children

## Abstract

**Objectives:** This study explored a multidimensional model of the relationships between social media use, gender, peer bullying victimization experiences, and the onset of anxiety symptoms among children. We hypothesized that greater experience of bullying would be associated with greater onset of anxiety. We also expected that gender and social media use (specifically Instagram and YouTube) would be linked with anxiety among elementary school children. To test this hypothesis, a structural equation modeling approach was used.

**Methods:** A total of 456 elementary children aged 11–13 years from nine schools were recruited for this research. We used two psychological measures: The Screen for Child Anxiety Related Emotional Disorders (SCARED) and the Personal Experience Checklist (PECK) as well as a sociodemographic questionnaire (general demographic information and social media-related information).

**Results:** The social media usage survey found that all participants (100%) used social media. Instagram (52.42%) and YouTube (47.58%) were the platforms most used by the participants. The Structural Equation Model results suggest that bullying victimization and gender predicted the onset of anxiety in elementary school children. The model explained 32.1% of the variance of the outcome with very adequate fit indicators based on most indices, χ^2^ = 173.56, df = 52, *p* < 0.001; CFI = 0.92; TLI = 0.94; RMSEA = 0.07 (90% CI: 0.06–0.08). Instagram use was correlated positively with generalized anxiety disorder. Gender was negatively correlated with Instagram use and positively correlated with YouTube use. Girls were found to use Instagram more and boys were found to use YouTube more. It was also found that girls had higher scores onSCARED dimensions, except for school avoidance. Girls were more prone to onset of anxiety than boys, except for school avoidance, which was not related to gender. Boys were found to experience significantly more physical bullying than girls. On the other hand, girls were found to experience more panic disorder, generalized anxiety disorder, separation anxiety disorder, and social anxiety than boys.

**Conclusion:** This study found that bullying victimization significantly influences the onset of anxiety in children. Particular attention should be paid to cyberbullying in this context. This study also found a link between gender and anxiety—girls had a greater tendency to experience the onset of various types of anxiety, including panic disorder, generalized anxiety disorder, separation anxiety disorder, and social anxiety. Gender was also correlated with the form of bullying victimization. The findings of this study suggest that boys were more likely to experience physical bullying than girls. Interestingly, we found that Instagram use was significantly correlated with developing separation anxiety. In particular, children demonstrated school avoidance when experiencing cyberbullying. Limitations and future directions are discussed.

## Introduction

According to Internet World Stats (2020), released in September 2020, Asia is the region of the world with the greatest number of Internet users–2.5 billion people, amounting to 51.8% of total users of the Internet. Indonesia is the third-largest user of the Internet in the world (Internet World Stats, 2020).

Based on the results of a survey by the Indonesian Internet Service Providers Association (APJII) and the Indonesia Survey Center, the number of Indonesian Internet users in 2019–2020 was 196.7 million, meaning that around 73.7% of Indonesians were connected to the Internet; the Java province dominates, with 55.7% of the total users. Furthermore, the number of social media users in Indonesia is 106 million out of a total population of 262 million (Triastuti et al., 2017). Based on the APJII survey (2020), the number of children using social media from year to year is increasing in line with the increasing number of Internet users in Indonesia. In Indonesia in 2019, 25.2% of children aged 5–9 years and 66.2% of children aged 10–14 years were active Internet users [Indonesian Internet Service Providers Association (APJII), 2020]. The use of the Internet in schools and online learning requires that children be connected to the digital world. In the same year, the majority of Internet users in Indonesia accessed online media for education and school content [Indonesian Internet Service Providers Association (APJII), 2020]. The lifestyles of various age groups, including children, especially in their late childhood, have changed in recent years. In contrast to children a few decades ago, children today are leading increasingly sedentary lifestyles that involve greater time spent on computers and watching TV (Bidzan-Bluma and Lipowska, 2018; Jochimek and Łada, 2019; Cornelius et al., 2020). In the last 15 years, the Internet has grown very fast: 40% of the world's population use the Internet, and the population of children is no exception. This generation is called the post-millennial and digital native generation because they are growing up in a wireless, hyper-networked environment that prefers communication over the Internet (Holton and Fraser, 2015). The reasons children use the Internet and social media include seeking information, to connect with friends (old and new), and for entertainment (Kominfo, 2014; Supratman, 2018).

This group spends 41% of their time in front of screens (Molter, 2020) and likes to share photos and videos via Instagram, YouTube, and Snapchat (Dolot, 2018). Furthermore, the types of social media most frequently used by children of the digital native generation are YouTube, Instagram, WhatsApp, FB, and Twitter (We Are Social, 2017; Supratman, 2018). In addition, Triastuti et al. (2017) noted that YouTube and Instagram are the most popular social media for children and adolescents. Furthermore, Triastuti et al. (2017) identified the following reasons for this group favoring these platforms, among others: their peers use these platforms; they have features that allow users to monitor each other, leave comments, share information (post daily activities), and communicate (message features); and entertainment.

Digital technology can positively impact children and adolescents in many ways, such as by improving their literacy and math skills, increasing socialization skills, by providing intellectual benefits such as problem-solving and critical thinking skills, as well as increasing imagination, art, and modeling skills (Undiyaundeye, 2014). Social media such as Facebook, Twitter, and YouTube can expand social connections and learning opportunities (McDool et al., 2016) and provide opportunities to connect with peers and for self-development (Ólafsson et al., 2013; Dyer, 2018). However, the Internet is a double-edged sword: Research has noted the negative impacts of digital technology on children, including poor self-esteem, mental health problems, and social difficulties (Wood et al., 2016). The use of social media, such as Instagram and YouTube, is also one of the leading causes of self-harm (McDool et al., 2016), cyberbullying, poor body image, and decreased academic performance (Wallsten, 2013; Akram and Kumar, 2017). Excessive use of social media generates anxiety (Sagioglou and Greitemeyer, 2014), addiction (Van Rooij and Prause, 2014), and can affect sleep behavior (Hisler et al., 2020). The increasing use of social media by children exposes them to various forms of bullying. This has increased the likelihood of children being perpetrators or victims, with multiple models or types of cyberbullying, ranging from gossip, ridicule, or coercion to bullying or violence from friends through electronic media. Some studies have documented the forms of bullying experienced by elementary school students through cyberspace or social media, for example, receiving unpleasant, nasty, or threatening text messages or emails (Noret and Rivers, 2006; Smith et al., 2008). Other studies have found that more students self-report having experienced traditional bullying than cyberbullying (Olweus and Limber, 2017; Bleam, 2018). Both forms of bullying contribute to emotional difficulties experienced by elementary school students. Cyberbullying is defined as violence, threats, coercion, or attempts to aggressively harass, humiliate, intimidate, or dominate another person using electronic media. It usually manifests as repetitive and hostile behavior shown by groups or individuals (Chatzakou et al., 2017). Previous research has considered the dimensions of cyberbullying from multiple perspectives. For example, Griezel et al. (2008) observed that traditional bullying consists of physical, verbal, and social dimensions, whereas cyberbullying consists only of visual and textual dimensions. Abeele and Cock (2013) reviewed previous research and identified two different types of cyberbullying: direct virtual bullying of the victim (e.g., sending threatening messages directly to the victim) and indirect or relational cyber bullying (e.g., gossiping without the victim's knowledge). Furthermore, Qing (2015) suggests that new forms of technology pose further challenges for defining cyberbullying. Inconsistencies in study results can lead to inaccurate estimates of the prevalence of cyberbullying. Some studies have found that there is an increase in the type of bullying from traditional bullying to cyberbullying as children aged (early to mid-adolescent students; Ortega et al., 2009; Charalampous et al., 2018). It is important to watch out for technological developments that increase the likelihood of students being bullied beyond what happens in schools, namely, in the cyber world or virtual space. Students' actions in this virtual space are often overlooked and are difficult for adults to monitor (Diamanduros et al., 2008; Rideout et al., 2010). Unsurprisingly, students who use the Internet are at risk of having experienced at least some virtual bullying (Smith et al., 2008; Kowalski et al., 2014).

Studies have reported adverse effects of bullying on children and adolescents (Lamarche et al., 2007; Merrell et al., 2008; Cardoos and Hinshaw, 2011; Cornell and Mehta, 2011). Victims of bullying tend to experience increased anxiety, depression, socio-emotional problems, low self-esteem, feelings of social inadequacy, behavioral difficulties, impaired academic performance, school avoidance, absenteeism, and increased dropout rates. Moreover, students with high social anxiety show poorer adjustment to school, manifesting avoidance behaviors in response, thereby contributing to them underperforming and putting them at risk of prematurely leaving the educational system (Delgado et al., 2019). A meta-analysis by Cunningham et al. (2015) identified a link between childhood bullying and psychotic symptoms. Stapinski et al. (2014) also found that adolescents who experienced bullying in childhood were two to three times more likely to have anxiety disorders.

In addition, Calleja and Rapee (2020) found an association between adolescents who self-reported victimization by their peers and internalizing symptoms (i.e., anxiety and depression). Adolescents who experience anxiety or depression are more sensitive to social threats when this correlates with self-reported victimization by their peers. Other studies have shown that anxious and depressed adolescents consistently interpret ambiguous social stimuli as threats and social rejection (Miers et al., 2008).

Furthermore, students with high social anxiety exhibit greater school absenteeism, are more stressed by academic tasks (Van-Roy et al., 2009), and are involved in fewer extracurricular activities than students without social anxiety (Delgado et al., 2019). Research conducted in several provinces in Indonesia shows that elementary school children in Yogyakarta province experience moderate anxiety (Naen, 2019) and that as many as 48% of elementary school children in the city of Bogor experience anxiety, and 84.7% experience depression (Utami et al., 2019).

In addition, gender differences have been found to have an effect on social media use. Mazman and Usluel (2011) identified that it is mostly men who use Facebook to make new friends, while it is mostly female users who use Facebook to maintain existing relationships, for academic purposes, and to pursue specific agendas. In addition, women are more selective when adding friends on social media than men (Kasahara et al., 2019). A study conducted by Booker et al. (2018) showed that the use of social media in girls at the age of 10 has the potential to reduce psychological well-being compared with boys.

In this study, we hypothesize that social media, bullying victimization, and gender will predict the onset of anxiety. Also, this research examines the strength and direction of the relationships of social media, bullying victimization, and gender with the onset of anxiety among Indonesian elementary school children.

## Materials and Methods

### Participants

A total of 456 elementary students from six grades, aged 11–13 years old (*M* = 11.17, *SD* = 0.43), from nine schools (five schools in Semarang, Central Java; four in Yogyakarta) were recruited for this research (52.41% of whom were male). These schools are located in the city center, and the students have adequate Internet access both at school and home. The students completed paper-and-pencil surveys in their classrooms during a single class period (50 min) under the supervision of trained research assistants (Table 1).

**Table 1:** Participant demographics.

**Aspect**	**Frequency (*****N*** **=** **456)**	
	***n***	**%**
**Gender**		
Male	239	52.41%
Female	217	47.58%
**Age**		
10	9	1.97%
11	356	78.07%
12	88	19.29%
13	3	0.65%

### Research Instruments

This study used two instruments: The Screen for Child Anxiety Related Emotional Disorders (SCARED) and the Personal Experience Checklist (PECK).

The SCARED was first developed by Birmaher et al. (1997). It measures anxiety disorders in children and adolescents and consists of 41 question items with five dimensions: 10 items concerning Panic Disorder or Significant Somatic Symptoms (e.g., *when I get frightened, I feel like passing out*); nine items concerning Generalized Anxiety Disorder (e.g., *I worry about other people liking me*); eight items concerning Separation Anxiety Disorder (e.g., *I get scared if I sleep away from home*); seven items concerning Social Anxiety Disorder (e.g., *I feel nervous with people I don't know well*); and four items concerning School Avoidance (e.g., *I get stomach aches at school*; Birmaher et al., 1999). The SCARED uses a Likert scale that ranges from 0 (*never*) to 2 (*frequently*). The scale is interpreted by adding up scores on all items: a score of more than 25 indicates the presence of an anxiety disorder. The reliability coefficient for panic disorder or significant somatic symptoms was 0.744, generalized anxiety disorder was 0.886, separation anxiety disorder was 0.76, social anxiety disorder was 0.732, and school avoidance was 0.752. The SCARED reliability coefficient in this study was 0.878.

The PECK is used to explore bullying and cyberbullying experienced by children and adolescents. It consists of 32 question items and four factors: relational–verbal bullying (e.g., *other kids say mean things behind my back*); cyberbullying (e.g., *other kids threaten me over the phone*); physical bullying (e.g., *other kids tell people to hit me*); and bullying based on culture (e.g., *other kids tease me about my accent*; Hunt et al., 2012). Relational–verbal bullying focuses on all forms of verbal abuse, for example, being called mean names, being made fun of, or being teased in a hurtful way (Hunt et al., 2012). Cyberbullying is negative behaviors that take place on mobile phones and the Internet, such as threats, spreading rumors, and being malicious (Grigg, 2010). Physical bullying is direct physical violence, for example, kicking or damaging the victim's property (Fu et al., 2015). Bullying based on culture occurs due to cultural factors, including differences in skin color, country of origin, culture, and/or religion (Rodríguez-Hidalgo et al., 2019). Respondents were asked to answer each question on a five-point Likert scale: 1 (*never*), 2 (*rarely*), 3 (*sometimes*), 4 (*once a week*), and 5 (*almost every day*). The total of all the respondents' answers indicates the individual's experience of bullying and cyberbullying and can be sorted into five categories: *not at all, somewhat bad, bad, very bad*, or *terrible*. The reliability coefficient of the PECK in this study was 0.889, and the subscale's reliability coefficient for relational–verbal bullying was 0.821, for cyberbullying was 0.762, for physical bullying was 0.765, and for bullying based on culture was 0.849.

### Data Collection Process

This study was approved by the Research Ethics Committee of the first author's institution and complied with the ethical standards for research involving human subjects. Before the survey was administered, students were provided with consent forms.

Participants were elementary school children. Based on predetermined age criteria, schools selected classes that could be accessed by researchers. In the early stages, the researchers conducted a meeting with the school and the parents/guardians in which they explained the study. The parents were then asked to provide their informed consent to the school within a maximum period of 1 week. Parents/guardians who gave their consent continued with the data collection process. All parents contacted agreed to give consent for the study.

They were fully informed that participation is voluntary, and they could either refuse to participate or withdraw from the study. Students' answers were confidential.

### Social Media Usage Survey

The social media usage survey showed that all participants were social media users. Instagram (52.42%) and YouTube (47.58%) were the primary platforms used by participants. More than half of the participants (56.79%) reported that the time spent on social media each day was 1–3 h. Detailed results of this survey can be found in Table 2.

**Table 2:** Social media usage survey.

**Aspect**	**Frequency (*****N*** **=** **456)**	
	***N***	**%**
**Do you use social media?**		
Yes	456	100%
**Social media platform**		
Instagram	239	52.42%
YouTube	217	47.58%
**Length of time spent on social media each day (hours)**		
1–3	259	56.79%
4–6	62	13.59%
>6	135	29.60%

### Data Analysis

All variables were screened for data entry accuracy, missing values, multivariate outliers, normality, linearity, and homoscedasticity. The association between continuous variables was tested utilizing Pearson correlations. Structural equation modeling (SEM) was used to test the hypothesized model. The study followed the two-step approach to SEM (Kline, 2005): first testing the measurement model to establish a statistically reliable measure for each construct and then testing the structural model to examine the multivariate relationships among the constructs. Model fit was assessed with the chi-square goodness-of-fit statistic (nonsignificant chi-square value), χ^2^/*df*, comparative fit index (CFI), where values above 0.90 indicate good fit and root mean square error of approximation (RMSEA) values <0.8 (Tabachnick and Fidell, 2001) or 0.05 indicate a “close fit” (Kline, 2005). These indices were used to evaluate whether the estimated covariance matrix was an adequate representation of the sample covariance matrix. All maximum likelihood estimations for the model were computed using IBM SPSS AMOS 24.0.

## Results

### Descriptive Statistics and Relations Among the Variables

Table 3 presents the means and standard deviations of the variables assessed, and Table 4 displays the matrix of correlations among social media types, gender, and PECK and SCARED dimensions. In the analyses, gender was made a dummy variable. Instagram use was correlated positively with generalized anxiety disorder. Gender was negatively correlated with Instagram use and positively with YouTube use. Girls were found to use Instagram more, and boys were found to use YouTube more. Additionally, it was also found that girls scored higher on SCARED dimensions, except for school avoidance. Girls were more prone to onset of anxiety than boys, except for school avoidance, which was not related to gender. Boys were found to experience significantly more physical bullying than girls. On the other hand, girls were found to experience more panic disorder, generalized anxiety disorder, separation anxiety disorder, and social anxiety than boys.

**Table 3:** Mean and standard deviations of bullying victimization and anxiety (*N* = 456).

	**Mean**	**Standard deviation**
**PECK**		
Relational–verbal Bullying	7.71	5.57
Cyberbullying	1.09	1.92
Physical bullying	5.56	4.25
Bullying based on Culture	1.07	1.41
**SCARED**		
Panic disorder	5.13	3.41
Generalized anxiety disorder	6.28	3.05
Separation anxiety disorder	6.57	2.91
Social anxiety	5.47	2.94
School avoidance	1.46	1.24

**Table 4:** Correlation matrix of social media types, gender, bullying victimization, and anxiety (*N* = 456).

	**1**	**2**	**3**	**4**	**5**	**6**	**7**	**8**	**9**	**10**	**11**	**12**
(1). Instagram	1											
(2). YouTube	−0.59^**^	1										
(3). Gender (boy = 1, girl = 0)	−0.21^**^	0.24^**^	1									
**PECK**												
(4). Relational–verbal bullying	−0.04	0.05	−0.06	1								
(5). Cyberbullying	0.03	−0.08	0.03	0.35^**^	1							
(6). Physical bullying	−0.04	0.06	0.10^*^	0.71^**^	0.48^**^	1						
(7). Bullying based on culture	0.01	−0.05	0.07	0.48^**^	0.27^**^	0.44^**^	1					
**SCARED**												
(8). Panic disorder	0.04	−0.02	−0.14^**^	0.44^**^	0.09	0.29^**^	0.22^**^	1	1			
(9). Generalized anxiety disorder	0.06	−0.04	−0.20^**^	0.42^**^	0.13^*^	0.27^**^	0.22^**^	0.63^**^	1			
(10). Separation anxiety disorder	0.10^*^	−0.03	−0.23^**^	0.29^**^	0.09	0.18^**^	0.09	0.53^**^	0.51^**^	1		
(11). Social anxiety	0.07	−0.03	−0.24^**^	0.22^**^	0.04	0.11^*^	0.04	0.40^**^	0.52^**^	0.42^**^	1	
(12). School avoidance	0.02	0.01	−0.08	0.31^**^	0.14^**^	0.19^**^	0.16^**^	0.38^**^	0.32^**^	0.33^**^	0.26^**^	1

** *Correlation is significant at the 0.01 level (two tailed)*.

* *Correlation is significant at the 0.05 level (two tailed)*.

The PECK items were all highly and positively correlated with each other. SCARED domains also showed moderate and positive relationships among each other. Relational–verbal bullying and physical bullying were positively correlated with all SCARED dimensions. Cyberbullying was positively correlated with general anxiety disorder and school avoidance domains; it was also significantly correlated with bullying based on culture, panic disorder, generalized anxiety disorder, and school avoidance.

**Figure 1:**
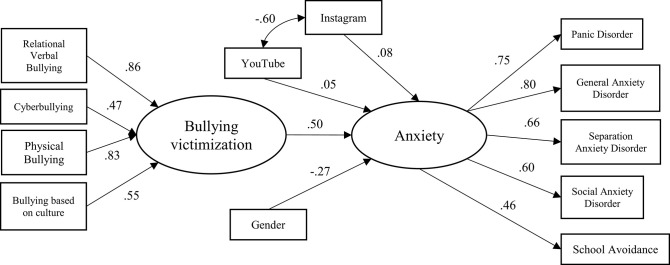
Structural equation model between variables.

Figure 1 presents the structural equation model results, suggesting that bullying victimization and gender predicted the onset of anxiety in elementary school children. The figure includes all the path coefficients that are significant at and beyond the 0.05 level. Based on the model and Table 5, anxiety was directly influenced by bullying victimization (β = 0.50) and gender (β = −0.27). The model explained 32.1% of the variance with very adequate fit indicators based on most indices, χ^2^ = 173.56, df = 52, *p* < 0.001; CFI = 0.92; TLI = 0.94; RMSEA = 0.07 (90% CI: 0.06–0.08).

**Table 5:** Maximum likelihood estimates of the model.

**Path**	**Unstandardized estimate**	**Standardized estimate**	**Standard errors**	**Critical ratio**
Bullying victimization → anxiety	0.27	0.50	0.03	8.96
Instagram → anxiety	0.45	0.08	0.32	1.38
YouTube → anxiety	0.28	0.05	0.29	0.95
Gender → anxiety	−1.37	−0.27	0.25	−5.54

### Gender Differences in Bullying Victimization and Anxiety

Table 6 presents the differences between boys and girls in terms of several aspects of bullying victimization and anxiety. There were significant differences in physical bullying, panic disorder, generalized anxiety disorder, separation anxiety disorder, social anxiety disorder, and school avoidance.

**Table 6:** Differences between genders.

		***N***	**Mean**	***t***
Relational–verbal bullying	Girls	217	8.078	1.354
	Boys	239	7.372	1.358
Cyberbullying	Girls	217	1.083	−0.074
	Boys	239	1.096	−0.074
Physical bullying	Girls	217	5.111	−2.144^**^
	Boys	239	5.962	−2.170^**^
Bullying Based on Culture	Girls	217	0.963	−1.492
	Boys	239	1.159	−1.508
Panic disorder	Girls	217	5.636	3.039^**^
	Boys	239	4.674	3.044^**^
Generalized anxiety disorder	Girls	217	6.926	4.417^**^
	Boys	239	5.686	4.401^**^
Separation anxiety disorder	Girls	217	7.272	5.032^**^
	Boys	239	5.933	5.025^**^
Social anxiety disorder	Girls	217	6.203	5.176^**^
	Boys	239	4.816	5.181^**^
School avoidance	Girls	217	1.567	1.777^*^
	Boys	239	1.360	1.774^*^

** *Difference is significant at the 0.01 level (two tailed)*.

* *Difference is significant at the 0.05 level (two tailed)*.

Boys tended to experience more physical bullying than girls. On the other hand, girls tended to experience more panic disorder, generalized anxiety disorder, separation anxiety disorder, social anxiety, and school avoidance than boys.

## Discussion

This study found that bullying victimization significantly affected anxiety onset in children. Being a victim of bullying was associated with several mental health issues, such as poor self-esteem, depression and anxiety, externalizing disorders, and even suicidal behavior, especially in girls. Bullying was closely related to externalizing disorders such as ADHD, conduct disorders, and oppositional defiant disorder. A meta-analysis by Cunningham et al. (2015) found that bullying in childhood is related to the development of psychotic symptoms. Copeland et al. (2013) found that students who are victims of bullying have higher prevalences of generalized anxiety disorder, panic disorder, and antisocial personality disorder than perpetrators. The relationship between social problems and the onset of panic attacks begins with poor social skills and difficulties in peer relationships, which in turn can lead to lower self-esteem and feelings of lack of control and helplessness (Mathyssek et al., 2012). Research in Indonesia has found that school-age children are very susceptible to bullying because this is the time at which children start to move out of the family environment and to mix and interact with peers (Wakhid et al., 2017). Peer groups play a large role in the school environment, as children spend most of their time with peers. As children want to be liked by their friends generally, they will do whatever their peers tell them to in order to be accepted by the group. Bullying in peer groups can often result in anxiety, loneliness, decreased sense of security, fear, depression, poorer school grades, and even running away from home. The onset of anxiety can be one significant effect of bullying, especially if the perpetrators are peers who should be a source of support and help meet the victim's socialization needs (Xie and Ngai, 2020).

Furthermore, this study also found that Cyberbullying was significantly correlated with generalized anxiety disorder, and school avoidance. This is in line with previous studies (Xantus et al., 2015) that found that children who experience significant bullying victimization have higher generalized anxiety scores. This can be due to such children feeling that school and school friendships are not a safe environment. In Indonesian culture, negative stigma from one's environment is considered to be very shameful (Budirahayu et al., 2018), so the relationship between friends is very important, in some cases, even greater than other needs (Lubis et al., 2019). An Indonesian study by Waliyanti and Kamilah (2019) found that teenagers can have high tolerances for acts of violence committed against their friends because bullies tend to dominate peer groups, causing teenagers to fear that they themselves will be bullied if they speak up. This can lead to the emergence of excessive anxiety among victims of bullying, including concerns about themselves experiencing acts of violence from others in their environment and the prospect of pain in the future (Pontillo et al., 2019). Thus, it is important that teachers provide a safe classroom environment and clearly communicate to students that bullying is a negative behavior that must be avoided, even though the perpetrator may be a member of their peer group.

Cyberbullying increases feelings of isolation and helplessness (Wang et al., 2012). Children who experience cyberbullying have fewer friends (Price and Dalgleish, 2010) and experience emotional and peer relationship problems (Sourander et al., 2015; Nicolai et al., 2018) as well as increased social anxiety (Dempsey et al., 2009; Fredstrom et al., 2011). Some studies (Randa, 2013; Randa and Reyns, 2014) have found that cyberbullying victimization positively correlates with fear of victimization in school and adaptive avoidance behavior at school.

This study also found that gender significantly affected anxiety onset and that boys experience more physical bullying than girls. The literature on bullying reveals that boys are more prone to be bullies and victims of bullying, especially physical bullying. At the same time, girls are more likely to be involved in emotional or indirect bullying, such as teasing or spreading falsehoods about their peers (Carbone-Lopez et al., 2010; Romera Félix et al., 2011; Clarke et al., 2012; AlBuhairan et al., 2017). This can be related to differences in characteristics between genders. The stereotypical characteristics of masculinity are closely related to acts of intimidation and violence, while feminine characteristics are related to victimization and verbal forms of aggression (Iossi Silva et al., 2013). In Indonesian culture, parents tend to instill the spirit of “*Kesatria*” in boys, represented by strong personal characteristics and a strong and dignified physical appearance, so that strength and physical appearance have a distinct value for boys (Fitri and Waluyo, 2019).

In line with this, indirect forms of bullying that often occur in boys are related to testing physical strength as an indication of dominance and power over other individuals (Iossi Silva et al., 2013). Bullies tend to feel proud and strong after “defeating” their “opponents” (Iossi Silva et al., 2013).

Interestingly, we found that using Instagram was significantly correlated with the onset of separation anxiety. Depressive symptoms, adverse moods, low self-esteem, and anxiety are marginally positively associated with Instagram (Lup et al., 2015; Marengo et al., 2018; Sherlock and Wagstaff, 2018). Furthermore, one longitudinal study (Vannucci and Ohannessian, 2019) found that children who frequently use Instagram are more likely to manifest delinquent and school avoidance behavior. Many school-age children learn about Instagram and then become active users based on the influence of people around them, including their parents, family members other than their parents (e.g., older siblings), and friends (Kurnia et al., 2017). They become active and enjoy sharing photos, commenting, and getting to know lots of new friends. However, it is very unfortunate that children can lack the technical skills, knowledge, and emotional maturity to navigate social media like Instagram, making them vulnerable to its negative effects.

### Limitations and Future Directions

No study is without limitations. All study variables were individual level, and further work is required across various levels of data. The study was carried out in a limited geographical area, Java Province, the largest in Indonesia, which has greater access to technology and facilities than other provinces. Consequently, the generalizability of results to different contexts is limited. Future investigations should explore and include the type and quality of schools, parental education level, SES indicators, and comparisons with the parents' perspective. Qualitative research could help obtain in-depth data regarding children's activity on Instagram and the experience of bullying and its effect on anxiety onset in relation to their social media behaviors. Our study also identifies the need to develop interventions and prevention strategies aimed at bullying victimization in children who use social media, such as teaching children digital literacy skills, digital resilience skills, and steps that can be taken when faced with situations that could potentially lead to bullying. Another strategy would be to develop programs that involve the active participation of schools and parents in creating a positive digital environment for children.

## Conclusion

This study found that bullying victimization significantly influenced onset of anxiety in children. Particular attention should be paid to cyberbullying in this context. This study also found a link between gender and anxiety—girls had a greater tendency to experience the onset of a variety of types of anxiety, including panic disorder, generalized anxiety disorder, separation anxiety disorder, and social anxiety. Gender was also correlated with the form of bullying victimization, with boys being more likely to experience physical bullying than girls. Interestingly, we found that Instagram use was significantly correlated with developing separation anxiety and that children demonstrated school avoidance when experiencing cyberbullying. However, further investigation is needed to explain the underlying psychological dynamics. The data presented in this paper on elementary school children who use social media can provide crucial insights for those who take care of them, namely, parents/guardians, teachers, and the government. These parties should pay careful attention to the digital well-being of elementary school children, for example through mentoring and social media literacy, to prevent bullying and, by extension, anxiety.

## Data Availability Statement

The raw data supporting the conclusions of this article will be made available by the authors, without undue reservation.

## Ethics Statement

The studies involving human participants were reviewed and approved by Faculty of Psychology, Diponegoro University, Semarang, Indonesia. Written informed consent to participate in this study was provided by the participants' legal guardian/next of kin.

## Author Contributions

DK conceptualized the study, acquired the funding, and analyzed the data. DK, RM, IK, and MB made substantial and direct intellectual contributions to the work, provided feedback, and approved the final version of the manuscript. All authors contributed to the article and approved the submitted version.

## Conflict of Interest

The authors declare that the research was conducted in the absence of any commercial or financial relationships that could be construed as a potential conflict of interest.
